# Management of Ulcerative Colitis Flare-Ups in the Era of COVID-19

**DOI:** 10.7759/cureus.32153

**Published:** 2022-12-03

**Authors:** Sara Mounsif, Hiba Setouani, Anass Nadi, Fahd Ghalim, Hanane Delsa

**Affiliations:** 1 Gastroenterology and Hepatology, Cheikh Khalifa International University Hospital, Mohammed VI University of Health Sciences (UM6SS), Casablanca, MAR

**Keywords:** coronavirus disease, inflammatory bowel disease, flare-up management, covid 19, flare-up, ulcerative colitis (uc)

## Abstract

Ulcerative colitis (UC) is a chronic inflammatory bowel disease (IBD) whose management depends on its severity, localization, and course. The coronavirus disease 2019 (COVID-19) pandemic has made the management of this disease more difficult, as severe acute respiratory syndrome coronavirus 2 (SARS-CoV-2) routinely causes respiratory infection, but can also target the gastrointestinal tract. Multiple cases of de novo IBD, IBD flare-ups, and colitis have been associated with COVID-19 infection. We present the case of two patients, with a history of UC, who presented respectively a mild and a severe flare-up of their disease associated with COVID-19 infection.

Regardless of recommendations, we decided to optimize the patient's treatment and obtained good clinical, biological, and endoscopic results. This report on the two cases suggests that remaining cautious and optimizing can be a good therapeutic alternative for these patients rather than modifying the treatment.

## Introduction

Ulcerative colitis (UC) is a chronic inflammatory bowel disease (IBD) whose annual incidence is estimated at 5-15 new cases per 100,000 inhabitants [1]. It evolves by flare-ups interspersed with phases of remission, with main symptoms including mucous and bloody diarrhea, abdominal pain, tenesmus, and asthenia.

The management of the disease depends on its severity, localization, and course. Various medical options exist and include 5-aminosalicylic acid (5-ASA) agents, corticosteroids, and immunosuppressive and biological agents in case of more extensive or severe disease [2].

In recent years, the coronavirus disease 2019 (COVID-19) pandemic has made the management of IBD patients more complicated, as it may cause a problem in differential diagnosis and in the management of these patients, especially when they present a flare-up of their disease associated with a COVID-19 infection.

We present the cases of two patients with a history of UC, who presented a flare-up of their disease associated with confirmed COVID-19 infection.

## Case presentation

Case 1

The first patient is a 35-year-old male, with a two-year history of UC on topical 5-ASA (1 g mesalazine rectal enema per week). He was admitted to our clinic during the first wave of COVID-19, in October 2020, for mucous and bloody diarrhea (10 stools per day), tenesmus, and profound asthenia. No respiratory symptoms were associated. Physical examination on admission found an asthenic patient with a tachycardic pulse (pulse rate: 110 per minute). His other vitals were normal (blood pressure: 110/70 mmHg, respiratory rate: 16 per min, temperature: 98.7 °F, and oxygen saturation: 100% on room air). 

The patient’s abdomen was tender but not rigid. Digital rectal examination was normal. Cardiovascular and pulmonary examinations were also normal. The patient’s flare-up was classified as severe according to the Truelove and Witts criteria [3]. He was hospitalized in the gastroenterology unit.

Investigations found a negative C-reactive protein, fecal calprotectin of 1025 μg/g (normal range: 0-120 µg/g), and *Entamoeba histolytica* cysts on parasitological stool examination. The severe acute respiratory syndrome coronavirus 2 (SARS-CoV-2) nasopharyngeal swab was positive. Chest CT was normal.

Metronidazole (1500 mg per day for two weeks) and corticosteroids (prednisolone 60 mg per day for one week) were administered. The dose of topical 5-ASA was increased to one rectal enema per day.

**Figure 1:**
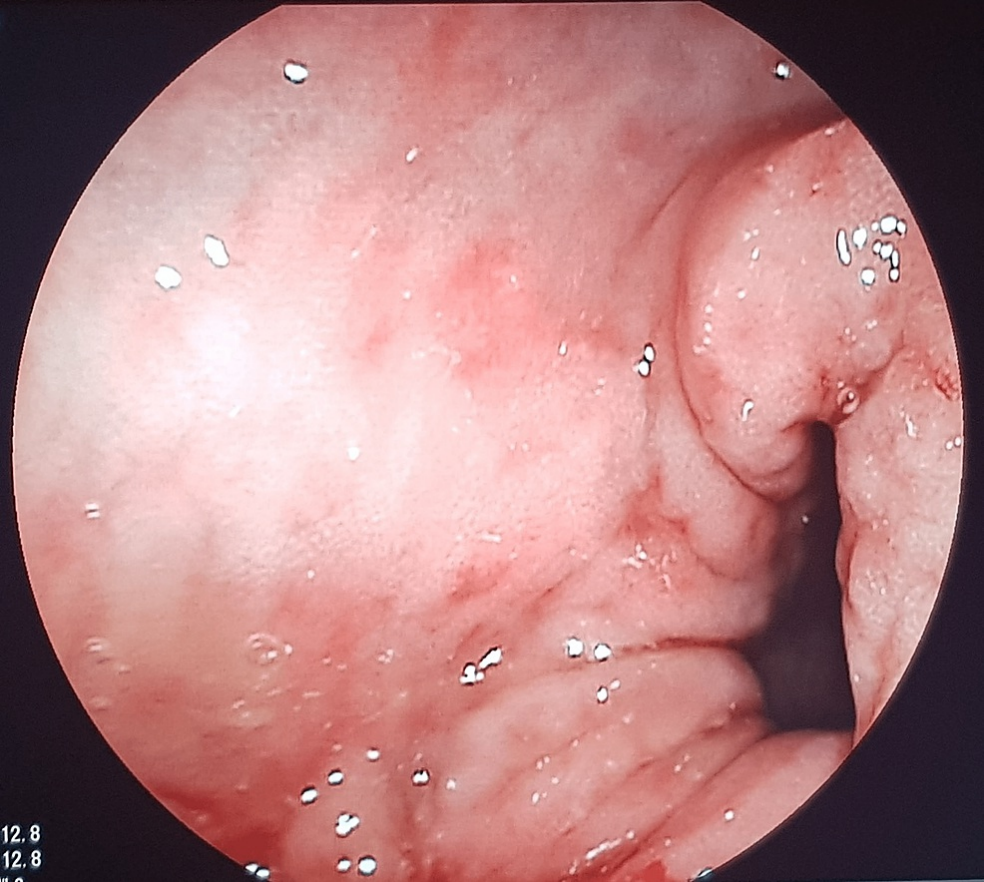
Endoscopic view of Mayo 1 proctitis Reduced vascular pattern with mucosal erythema

The next day, the patient showed improvement in his symptoms with only one to two stools per day. CT enterography showed rectal wall thickening likely of inflammatory origin. We decided to continue the same treatment and see the patient one month after his discharge. Control colonoscopy showed Stage 1 proctitis (Figure 1) according to Mayo classification [4], and fecal calprotectin was negative. Currently, two years later, the patient is in remission, and still on topical 5-ASA.

Case 2

The second patient is a 40-year-old female with an 11-year history of UC on maintenance with infliximab (5 mg/kg) and azathioprine (50 mg twice daily). In February 2021, she was admitted to our clinic for mucus and bloody diarrhea (three stools per day). One week before her admission, she presented with flu-like symptoms and a dry cough. Physical examination and vital signs were normal. The patient’s flare-up was classified as mild according to Truelove and Witts criteria.

Initial workup showed negative C-reactive protein and increased fecal calprotectin (1300 μg/g). The SARS-COV-2 nasopharyngeal swab was positive.

**Figure 2:**
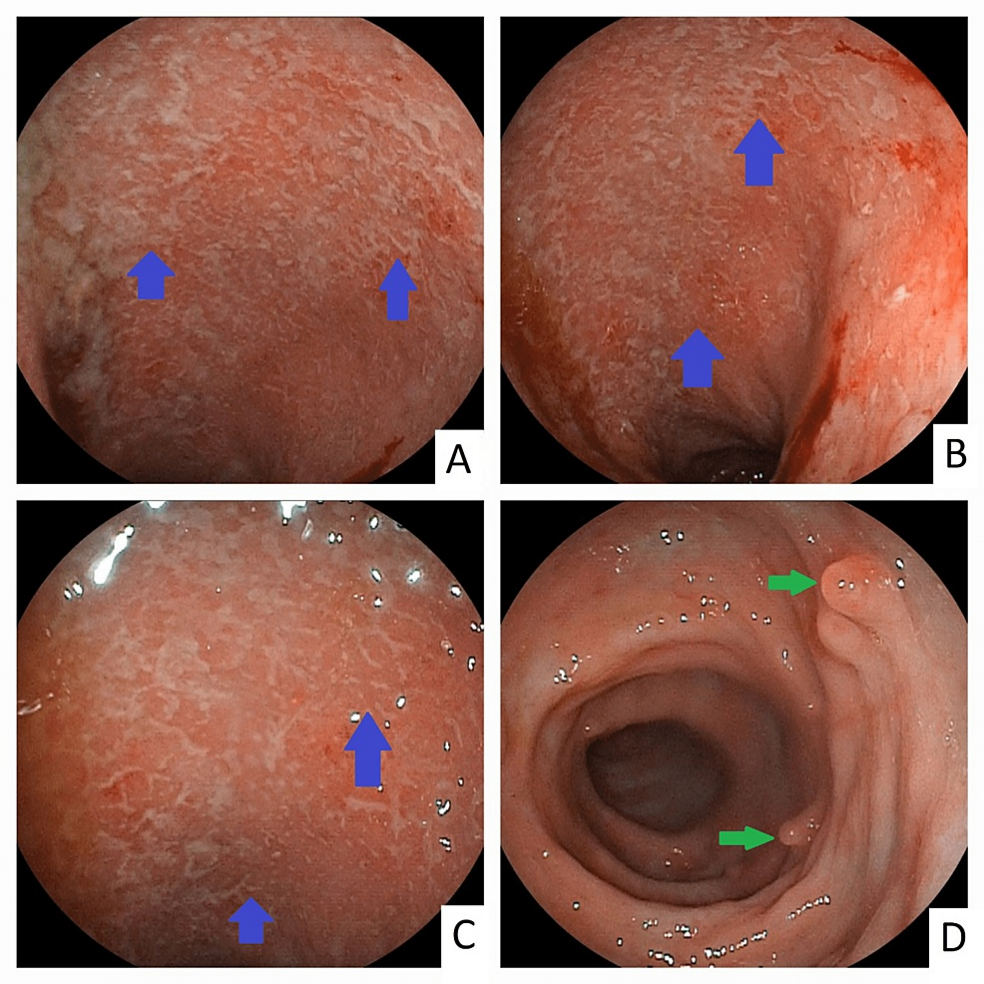
Endoscopic view of Mayo 3 proctosigmoiditis with significant mucosal granularity and ulcerations with spontaneous bleeding (blue arrows; panels A, B, and C) with multiple pseudopolyps (green arrows, panel D)

The patient was treated in ambulatory with 1 g 5-ASA rectal enema per day, with rapid improvement of symptoms. After two weeks, a colonoscopy was performed and showed Mayo 3 proctosigmoiditis with multiple pseudopolyps (Figure 2). Histological examination was consistent with evolving UC.

**Figure 3:**
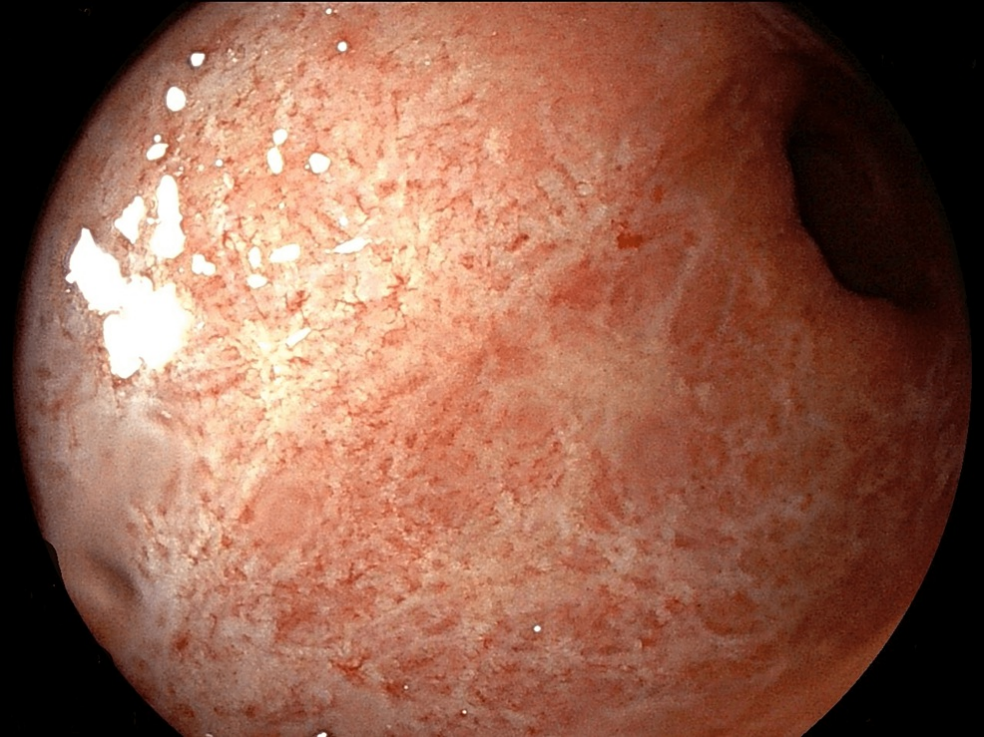
Endoscopy showing control of proctosigmoiditis

Topical 5-ASA was continued and the patient's next infliximab infusion was delayed for two weeks after recovery. At one month, endoscopic appearance improved (Figure 3), and fecal calprotectin was negative. One year later, the patient is still under the same infliximab protocol, with clinical and endoscopic remission.

## Discussion

COVID-19 is an infectious disease caused by SARS-CoV-2, a new strain of coronavirus. As on October 15, 2022, more than 600 million COVID-19 confirmed cases were reported in the world [5]. Symptoms are multiple and include dry cough, fever, asthenia, myalgia, ageusia, anosmia, nausea, vomiting, and diarrhea. Over time, multiple cases of colitis caused by SARS-CoV-2 gastrointestinal infection have also been described, with manifestations of severe abdominal pain and acute bloody diarrhea in some cases [6,7].

Recent studies have shown that COVID-19 infection is associated with the development of autoimmunity and can trigger multiple autoimmune diseases, ranging from organ-specific to systemic autoimmune and inflammatory diseases [8]. Multiple cases of de novo inflammatory bowel diseases following COVID-19 infection or vaccination have been reported [9,10]. As direct causation is difficult to prove, an association remains controversial. Therefore, the management of digestive symptoms in the case of COVID-19 can sometimes be difficult.

The differential diagnosis between gastrointestinal symptoms of COVID-19 infection, SARS-CoV-2-induced colitis or de novo IBD, and an IBD flare associated with COVID-19 infection can be problematic. Also, multiple concerns were raised about the safety of IBD medications, primarily azathioprine and biologic therapies, during the COVID-19 pandemic, as they often suppress the immune system and can be associated with an increased risk of severe infections [11]. However, most international groups recommend that patients with an established diagnosis of IBD should remain on their pre-pandemic IBD therapy that was successful in inducing and maintaining remission [12].

Although in the case of IBD associated with confirmed COVID-19 infection, experts recommend modification of IBD therapy, mostly to decrease oral corticosteroids or switch to budesonide and delay biological therapy for two weeks until recovery [13], in some cases, infliximab initiation in IBD patients with active disease and COVID-19 appeared to be effective in the improvement of both pulmonary and digestive symptoms [14].

In our case, our patients had a mild COVID-19 infection associated with mild and severe UC flare-ups. We decided to optimize the patient's treatment, mainly with topical 5-ASA, instead of intensifying or modifying their treatment. We obtained good clinical results with an improvement in their symptoms after one to two days and no endoscopic signs of severity.

However, the decision to optimize or modify the patient’s treatment must be discussed on a case-by-case basis and requires close monitoring of the patient, at least during the first week.

## Conclusions

The management of IBD flare-ups associated with COVID-19 infection remains controversial and challenging for healthcare workers. Through our patients, the optimization of the treatment with 5-ASA appears to be a satisfactory option in the management of UC flare-ups associated with mild COVID-19 infection. However, studies with a larger population are needed to confirm this hypothesis.
